# Epistatic interactions among multiple copies of *FLC* genes with naturally occurring insertions correlate with flowering time variation in radish

**DOI:** 10.1093/aobpla/plac066

**Published:** 2023-02-02

**Authors:** Yuki Mitsui, Hinano Yokoyama, Wataru Nakaegawa, Keisuke Tanaka, Kenji Komatsu, Nobuya Koizuka, Ayako Okuzaki, Takashi Matsumoto, Manabu Takahara, Yutaka Tabei

**Affiliations:** Faculty of Agriculture, Tokyo University of Agriculture, 1737 Atsugi, Kanagawa 243-0034, Japan; Faculty of Agriculture, Tokyo University of Agriculture, 1737 Atsugi, Kanagawa 243-0034, Japan; Faculty of Agriculture, Tokyo University of Agriculture, 1737 Atsugi, Kanagawa 243-0034, Japan; NODAI Genome Research Center, Tokyo University of Agriculture, 1-1-1 Sakuragaoka, Setagaya-ku, Tokyo 156-8502, Japan; Faculty of Agriculture, Tokyo University of Agriculture, 1737 Atsugi, Kanagawa 243-0034, Japan; College of Agriculture, Tamagawa University, 6-1-1 Tamagawa Gakuen, Machida, Tokyo 194-8610, Japan; College of Agriculture, Tamagawa University, 6-1-1 Tamagawa Gakuen, Machida, Tokyo 194-8610, Japan; Faculty of Applied Biology, Tokyo University of Agriculture, 1-1-1 Sakuragaoka, Setagaya-ku, Tokyo 156-8502, Japan; Institute of Agrobiological Sciences, National Agriculture and Food Research Organization, Tsukuba, Ibaraki, 305-8634, Japan; Faculty of Food and Nutritional Sciences, Toyo University, 1-1-1 Izumino, Itakura-machi, Ora-gun, Gunma 374-0193, Japan

**Keywords:** Allelic variation, epistatic interaction, *FLOWERING LOCUS C*, gene expression, late-bolting trait, vernalization

## Abstract

Brassicaceae crops, which underwent whole-genome triplication during their evolution, have multiple copies of flowering-related genes. Interactions among multiple gene copies may be involved in flowering time regulation; however, this mechanism is poorly understood. In this study, we performed comprehensive, high-throughput RNA sequencing analysis to identify candidate genes involved in the extremely late-bolting (LB) trait in radish. Then, we examined the regulatory roles and interactions of radish *FLOWERING LOCUS C* (*RsFLC*) paralogs, the main flowering repressor candidates. Seven flowering integrator genes, five vernalization genes, nine photoperiodic/circadian clock genes and eight genes from other flowering pathways were differentially expressed in the early-bolting (EB) cultivar ‘Aokubinagafuto’ and LB radish cultivar ‘Tokinashi’ under different vernalization conditions. In the LB cultivar, *RsFLC1* and *RsFLC2* expression levels were maintained after 40 days of cold exposure. Bolting time was significantly correlated with the expression rates of *RsFLC1* and *RsFLC2*. Using the EB × LB F2 population, we performed association analyses of genotypes with or without 1910- and 1627-bp insertions in the first introns of *RsFLC1* and *RsFLC2*, respectively. The insertion alleles prevented the repression of their respective *FLC* genes under cold conditions. Interestingly, genotypes homozygous for *RsFLC2* insertion alleles maintained high *RsFLC1* and *RsFLC3* expression levels under cold conditions, and two-way analysis of variance revealed that *RsFLC1* and *RsFLC3* expression was influenced by the *RsFLC2* genotype. Our results indicate that insertions in the first introns of *RsFLC1* and *RsFLC2* contribute to the late-flowering trait in radish via different mechanisms. The *RsFLC2* insertion allele conferred a strong delay in bolting by inhibiting the repression of all three *RsFLC* genes, suggesting that radish flowering time is determined by epistatic interactions among multiple *FLC* gene copies.

## Teaser

Understanding the mechanisms of flowering time regulation is important for plant biology and crop improvement. The genomes of the Brassicaceae crops, including *Brassica*(e.g. cabbage, turnip, rapeseed) and *Raphanus*(radish) are triplicated and their flowering may be regulated by interactions of replicated copies of flowering-related genes. This study revealed that the gene expression of the three copies of *RsFLC*genes mainly contributed to the late-flowering in radish. In particular, the continuous expression of *RsFLC2*conferred a strong delay in flowering, possibly by inhibiting the repression of *RsFLC1*and *RsFLC3*.

## Introduction

The transition from vegetative growth to bolting and flowering is a crucial stage of the plant life cycle. Bolting and flowering times must be appropriately regulated to ensure reproductive success during favourable conditions (Amasino and Michaels 2010; Srikanth and Schmid 2011); thus, there has been intense interest in determining the genetic architecture of this phenomenon (Fitter and Fitter 2002; Craufurd and Wheeler 2009; Schlenker and Roberts 2009; Li *et al.* 2010). In *Arabidopsis thaliana*, the transition to flowering is under strict endogenous and environmental control. Floral initiation is induced both externally (vernalization- and photoperiod-dependent) and internally (autonomously and gibberellin-dependent) (Koornneef *et al.* 1998; Simpson and Dean 2002), forming an integrated regulatory network that precisely controls the timing of floral transition in a given environment. Signals from these pathways are integrated by the floral integrator genes *FLOWERING LOCUS T* (*FT*) and *SUPPRESSOR OF OVEREXPRESSION OF CONSTANS1* (*SOC1*), which then promote floral transition via activation of downstream genes such as *APETALA1* (*AP1*) and *LEAFY* (*LFY*) (Yankovsky and Kay 2002). Among multiple seasonal cues, temperature plays an essential regulatory role, particularly in plants that require an extended cold period to initiate flowering (Sung and Amasino 2004). A key component of both the vernalization and autonomous regulatory networks in Brassicaceae plants is *FLOWERING LOCUS C* (*FLC*), a MADS-box transcription factor that quantitatively inhibits floral transition (Michaels and Amasino 1999; De Lucia *et al.* 2008). Floral integrator *FT* genes are expressed in leaves under long-day conditions and transported to the shoot apex, initiating floral transition (Huang *et al.* 2005; Parcy 2005). Under normal conditions, the FLC protein represses the expression of *SOC1*, an integrator of the bolting and flowering pathway, by binding to the CArG-box present in the *SOC1* promoter (Hepworth *et al.* 2002). However, *FLC* expression decreases during cold exposure, thereby triggering upregulation of *SOC1* and accelerating floral transition under long-day conditions (Michaels and Amasino 1999; Michaels *et al.* 2005; Searle *et al.* 2006). Epigenetic silencing of *FLC* plays a central role in the vernalization pathway. The cold-induced plant homeodomain (PHD) finger protein, VERNALIZATION INSENSITIVE 3 (VIN3), forms a heterodimer with a related PHD protein, VERNALIZATION 5 (VRN5), which induces the formation of a PHD-polycomb repressive complex 2 (PRC2) complex that associates with the 5ʹ region of *FLC* (Sung *et al.* 2006). This association promotes the distribution of VRN5 along the *FLC* gene and significantly increases the level of *FLC* histone 3 lysine 27 trimethylation (H3K27me3), which maintains *FLC* silencing after returning to warm conditions (Finnegan and Dennis 2007; De Lucia *et al.* 2008). The formation of the PHD–PRC2 complex and its interaction with *FLC* are also mediated by several long non-coding RNAs (lncRNAs) (Heo and Sung 2011; Kim and Sung 2017).

Variation in temperature-dependent flowering times is mainly a result of quantitative variation in the expression and silencing of the floral repressor gene *FLC*. *FL*C expression is regulated by several genes including *FRIGIDA* (*FRI*), which represses flowering by upregulating *FLC* (Michaels and Amasino 1999; Johanson *et al.* 2000). *FLC* expression is stably repressed during vernalization in response to prolonged cold exposure, which accelerates flowering; however, FRI upregulates *FLC* expression, thus preventing flowering (Michaels and Amasino 2001; Searle *et al.* 2006). Allelic variations in *FRI* and *FLC* account for much of the natural variation in flowering time in *Arabidopsis* (Shindo *et al.* 2005). Specifically, winter-annual *Arabidopsis* has dominant alleles of *FRI* and *FLC* and requires vernalization for flowering (Michaels and Amasino 2001), whereas many summer-annual and rapid-cycling ecotypes can flower rapidly without vernalization. Summer-annual plants have evolved on multiple occasions, via disruption of *FLC* regulatory sequences by transposons (Michaels *et al.* 2003) and loss of an active *FRI* allele, thus lowering *FLC* expression levels (Johanson *et al.* 2000). In *A. thaliana*, cold-induced repression of *FLC* involves *cis*-regulatory sequences in the promoter and first intron of *FLC* (Sheldon *et al.* 2002; Sung *et al.* 2006). Mutations in these regions cause variation in *FLC* expression, which is associated with flowering time variation. For example, a *MUTATOR*-like transposon (~1.3 kb) was discovered in the downstream region of the first intron of *FLC* in *A. thaliana* (Ler), which exhibits an early-flowering phenotype (Gazzani *et al.* 2003; Michaels *et al.* 2003; Wang *et al.* 2006). A homologous transposon (1.19 kb) was found in the upstream region of the first intron of *FLC* in two *A. thaliana* North American accessions, and this transposon contributes to the downregulation of *FLC* (Strange *et al.* 2011). A 557-bp deletion in the region upstream of the first intron of *FLC* in *A. thaliana/A. arenosa* allotetraploids was associated with downregulation of *FLC* (Wang *et al.* 2006). Furthermore, two insertions/deletions (indels) and a substitution in the first intron of *FLC* were associated with variation in flowering time and vernalization sensitivity in wild populations of *A. thaliana* on the Iberian Peninsula (Mendez-Vigo *et al.* 2011). Allelic variation in *Brassica rapa FLC* genes (*BrFLC1*, *BrFLC2*, *BrFLC3*, *BrFLC5*) is responsible for differences in flowering times (Yuan *et al.* 2009; Zhao *et al.* 2010; Kakizaki *et al.* 2011; Kitamoto *et al.* 2014). For example, an extremely late-bolting (LB) strain of *B. rapa* contains a naturally occurring large insertion (~5 kb) at the 5ʹ end of the first intron of *BrFLC2*, resulting in reduced *FLC* gene repression under cold conditions (Kitamoto *et al.* 2014). In *B. oleracea*, 49 polymorphisms, each differing from polymorphisms present in *AtFLC* haplotypes, were found in two *BoFLC.C2* genotypes with different vernalization sensitivities; these polymorphisms influenced *BoFLC* expression and vernalization sensitivity (Irwin *et al.* 2016). The vernalization response element (VRE) is located in the region upstream of the first intron of *FLC* and is essential for *FLC* regulation (Sung *et al.* 2006). The cold-induced PHD–PRC2 complex associates with the VRE in the *FLC* promoter after prolonged cold conditions, in which it maintains *FLC* silencing by increasing H3K27me3 levels throughout the gene. The VREs of several Brassicaceae species share homology with the *A. thaliana* VRE, indicating that they are evolutionary conserved (Castings *et al.* 2014).

Radish (*Raphanus sativus*) is a diploid species (2*n* = 2*x* = 18) from the family Brassicaceae and tribe Brassiceae, which includes economically important crops such as *B. rapa* and *B. oleracea*. Comparative genomics has suggested that genomes of the Brassiceae crops, including the diploid *Brassica* and *Raphanus* species, are triplicated and rearranged from the ancestral genome (Kitashiba *et al.* 2014; Mitsui *et al.* 2015; Jeong *et al.* 2016). In crops such as radish, the vegetative parts of the plant such as roots, stems and leaves are harvested; understanding the molecular mechanisms of flowering is important for cultivating these vegetables because the initiation of flowering suppresses vegetative growth, which in turn reduces the yield and quality of the harvested products. Transcriptome analyses revealed 142 radish genes that were orthologous to *A. thaliana* flowering genes (Nie *et al.* 2016a), indicating that radish flowering is regulated by pathways similar to those in *A. thaliana* and other Brassicaceae plants. Three paralogous *FLC* genes (*RsFLC1*, *RsFLC2* and *RsFLC3*) were discovered in radish by genome sequencing (Kitashiba *et al.* 2014; Jeong *et al.* 2016) and transcriptome analysis (Yi *et al.* 2014). Because replicated gene loci contribute to gene function diversification, the multiple *FLC* paralogs in radish provide a unique system for evaluating regulatory interactions among evolutionary divergent loci originating from orthologous genomes. Overexpression of each of the three radish *FLC* (*RsFLC*) genes in *A. thaliana* transgenic plants induced late flowering, suggesting that each *RsFLC* functions as a floral repressor (Yi *et al.* 2014). The flowering time of radish is regulated by the quantitative effects of the three *RsFLC* genes; however, the repression activity of each *FLC* and their epistatic interactions are currently unclear. Furthermore, there is great variation in vernalization sensitivities among radish cultivars. A comparison of the transcriptomes of the early-bolting (EB) radish cultivar, which requires 15 days of cold for vernalization, and the LB cultivar, which requires 35 days of cold for vernalization, revealed that 49 flowering time-related genes were differentially expressed, and that *FLC* expression was relatively high in the LB cultivar under cold conditions (Jung *et al.* 2016). However, only *RsFLC1* expression was analysed, and the mechanisms driving *RsFLC* expression variation, as well as the interactions among the three *FLC* genes, were not elucidated. A 1627-bp insertion in the region upstream of the first intron of *RsFLC2* was discovered in the extremely LB radish cultivar (requires more than 60 days of cold for vernalization) via quantitative trait locus (QTL) analysis using 500 indel markers; the insertion allele weakened *RsFLC2* repression under cold conditions (Wang *et al.* 2018b). Moreover, a 1910-bp insertion in the mid-region of the first intron of *RsFLC1* was found in several LB cultivars; cultivars carrying both the 1910-bp insertion in *RsFLC1* and 1627-bp insertion in *RsFLC2* require longer periods of cold for flowering (Takii Seed Co. 2015). These studies indicate that insertions in the first introns of *RsFLC1* and *RsFLC2* regulate *FLC* expression. To understand the effects of the two allelic variations on radish *FLC* regulation, the relationships among the genotypes, phenotypes and the variation in the expression of the three *RsFLC* genes must be elucidated.

In this study, the candidate genes and allelic variation responsible for extremely LB phenotypes were identified in radish. First, we performed transcriptome analysis to detect differentially expressed genes (DEGs) involved in flowering pathways between the EB cultivar ‘Aokubinagafuto’ and the extremely LB radish cultivar ‘Tokinashi’ under various vernalization conditions. We found that the expression patterns of flowering integrator genes, including three *RsFLC* genes, differed greatly between the EB and LB cultivars. Next, EB and LB cultivars were crossed to generate an F2 population, enabling segregation of *RsFLC1* and *RsFLC2* alleles with or without insertions in their first introns. We then evaluated the regulatory roles and interactions between *RsFLC1* and *RsFLC2* insertion alleles in late-flowering traits by analysing associations among the *RsFLC* genotypes, expression patterns of the three *RsFLC* genes and bolting times.

## Materials and Methods

### Plant materials

Throughout this study, we used the EB commercial radish cultivar ‘Aokubinagafuto’ (Fukukaen Nursery & Bulb Co., Nagano, Japan) and the extremely LB commercial radish cultivar ‘Tokinashi’ (Sakata Seed Co., Kanagawa, Japan). The seeds were sown in plastic pots with red clay soil. After germination, the seedlings were grown at 20 °C under a 16-h:8-h light:dark cycle for approximately 3 weeks. Then, seedlings with two or three leaves were transferred to cold conditions (5 °C) for 20, 40, 60 and 80 days under a 10-h:14-h light:dark cycle for vernalization (V20, V40, V60 and V80, respectively). After cold treatment, seedlings were cultivated at 20 °C under a 16-h:8-h light:dark cycle for 60 days to determine the bolting time for each cultivar under each condition.

### RNA extraction, cDNA synthesis and RNA-Seq analysis

Total RNA was extracted from three leaves of each individual plant using the NucleoSpin RNA Plant Kit (Macherey-Nagel, Düren, Germany). As 20–40 days of cold exposure is sufficient to induce bolting in the EB cultivar, whereas the LB cultivar does not bolt within this period, leaves were collected after 20 and 40 days of cold treatment (V20T0 and V40T10) and after 10 days of warm conditions following cold treatment (V20T10 and V40T10). Total RNA was also extracted from each seedling just before cold treatment (V0T20). Three biological replicates were examined for each condition.

RNA quality and quantity were assessed using the 2100 Bioanalyser and RNA 6000 Nano Kit (Agilent Technologies, Palo Alto, CA, USA). Using equal volumes of total RNA from each sample, cDNA libraries were prepared using the mRNA-Seq Sample Preparation Kit (Illumina, San Diego, CA, USA) according to the manufacturer’s instructions. Illumina RNA sequencing was performed using the HiSeq 2000 platform at the Nodai Genome Research Centre (NRGC, Tokyo, Japan) in accordance with the manufacturer’s instructions.

RNA-Seq raw reads were imported into the CLC Genomics Workbench v11, and the reads were trimmed using the default parameters. The 46 514-gene model from the radish genome database (Jeong *et al.* 2016) was used as a reference for mapping. Trimmed reads were mapped to reference sequences using the default parameters, and gene expression values were calculated. Differentially expressed genes were identified between all pairs of libraries using Student’s *t*-test. Comparisons among vernalization conditions and between cultivars were performed using analysis of variance (ANOVA), followed by Bonferroni correction. Differential expression was considered significant at *P* < 0.05.

A total of 142 flowering-related genes were previously identified in a study of the radish transcriptome (Nie *et al.* 2016a), and 132 and 280 genes, excluding and including paralogs, respectively, were found in the radish genome (Jeong *et al.* 2016). The 280 genes include 11 flowering integrator genes (three copies of *RsFLC*, two of *RsFT*, two of *RsLFY* and four of *RsSOC1*s), 63 vernalization genes, 119 photoperiod and/or circadian clock genes and 87 other genes (age, autonomous, flower development and genes of other flowering pathways). Differentially expressed genes among these 280 flowering-related genes were analysed.

### Quantitative reverse-transcription polymerase chain reaction analysis of the three *RsFLC* genes

The expression of the three *RsFLC* genes was validated by quantitative reverse-transcription polymerase chain reaction (qRT-PCR) using the KAPA SYBR FAST qPCR kit (Kapa Biosystems, Woburn, MA, USA). Because differences in vernalization response between the EB and LB cultivars were clearer under the V40 treatment, the relative expression levels of V0T20 and V40T10 were analysed by qRT-PCR using the same RNA samples as used for RNA-Seq (T10 and T20 indicate 10 and 20 days after the return to warm conditions, respectively). To investigate the cold period required for bolting in the LB cultivar, newly extracted RNAs from V60T0, V60T10, V80T0 and V80T10 samples were also analysed. The 20-μL reaction mixture contained 20 ng cDNA template, 10 μL 1× KAPA SYBR FAST qPCR master mix, 5 μM forward and reverse primers and PCR-grade water. Primer information is presented in **Supporting Information—Table S1**. Primers used for the flanking exonic regions were designed based on their genomic sequences (Jeong *et al.* 2016) using Primer 3 software (http://primer3.ut.ee/) (Rozen and Skaletsky 2000). Polymerase chain reaction was performed using the LightCycler 480 system (Roche, Basel, Switzerland) with the following cycling conditions: 10 min of incubation at 95 °C, followed by 50 cycles of 95 °C for 10 s, 60 °C for 10 s and 72 °C for 10 s, with a single fluorescence reading taken at the end of each cycle. Melt curve analysis was performed for all runs to confirm amplification specificity and the lack of primer dimers. Second-derivative values were calculated to estimate expression levels. The expression levels were normalized to those of the stably expressed housekeeping gene *CAM7* (Mitsui *et al.* 2015). Gene expression was quantified using the ΔΔCt method (Livak and Schmittgen 2001). Two technical replicates of each sample were analysed.

### F1 and F2 population generation and phenotyping

F1 hybrids were generated by crossing EB (paternal) and LB (maternal) cultivars after removing the stamen of the maternal LB flower. F2 seeds were obtained by randomly crossing 16 F1 individuals. Ten and 30 individuals of the F1 and F2 populations, respectively, were grown and vernalized following the same protocol applied to the parental strains, and bolting rates were measured. In addition, a total of 103 F2 seedlings was vernalized under 40 days of cold and grown for 60 days at 20 °C. The number of days to bolting after the end of cold treatment was recorded for each individual.

### F2 population *FLC* expression pattern genotyping and qRT-PCR analysis

Genomic DNA from the 103 F2 individuals was extracted from seedling leaves that were homogenized using a multi-bead shocker (Yasui-Kikai, Osaka, Japan) and the NucleoSpin Plant II Kit (Macherey-Nagel) according to the manufacturers’ instructions. Partial sequences of the first introns of *RsFLC1* and *RsFLC2* were amplified by PCR using KOD-Plus-Neo polymerase (Toyobo, Osaka, Japan). The 25-μL PCR mixture contained 20 ng DNA template, 2.5 µL 10× KOD-Plus-Neo PCR Buffer, 2.5 μL 2 mM dNTPs (final concentration, 0.2 mM each), 1.5 μL 25 mM magnesium sulfate (final concentration, 1.5 mM), 10 μM forward and reverse primers (final concentration, 0.15 mM each), 1.0 μL KOD-Plus-Neo polymerase (1 U µL^−1^) and PCR-grade water. Primer information is presented in **Supporting Information—Table S2**. The reaction was performed using the Bio-Rad T100 Thermal Cycler under the following PCR cycling conditions: 2 min of incubation at 94 °C, followed by 30 cycles of two-step cycling conditions (98 °C for 10 s; 68 °C for 2 min). The PCR products were analysed by 1.0 % agarose gel electrophoresis and genotyped for alleles with or without indels in the first introns of *RsFLC1* and *RsFLC2*.

For the 103 F2 individuals used for phenotyping and genotyping, total RNA was extracted from three leaves of each individual plant vernalized under the V40T10 treatment using the NucleoSpin RNA Plant Kit (Macherey-Nagel). The expression of the three *RsFLC* genes in the F2 population was analysed by qRT-PCR following the same protocol applied to the parental strains.

### Statistical analyses

Expression levels of the three *RsFLC* genes were compared among genotypes with or without the *RsFLC1* or *RsFLC2* insertion alleles using Kruskal–Wallis and Steel–Dwass tests, at a level of significance of 5 %. Statistical analyses were performed using one-way ANOVA or Student’s two-tailed *t*-test for paired comparisons using SPSS v23.0 software (SPSS Inc., Chicago, IL, USA). Statistical significance was evaluated at *P* ≤ 0.05.

## Results

### Bolting time in parent and hybrid populations

**Figure 1.**
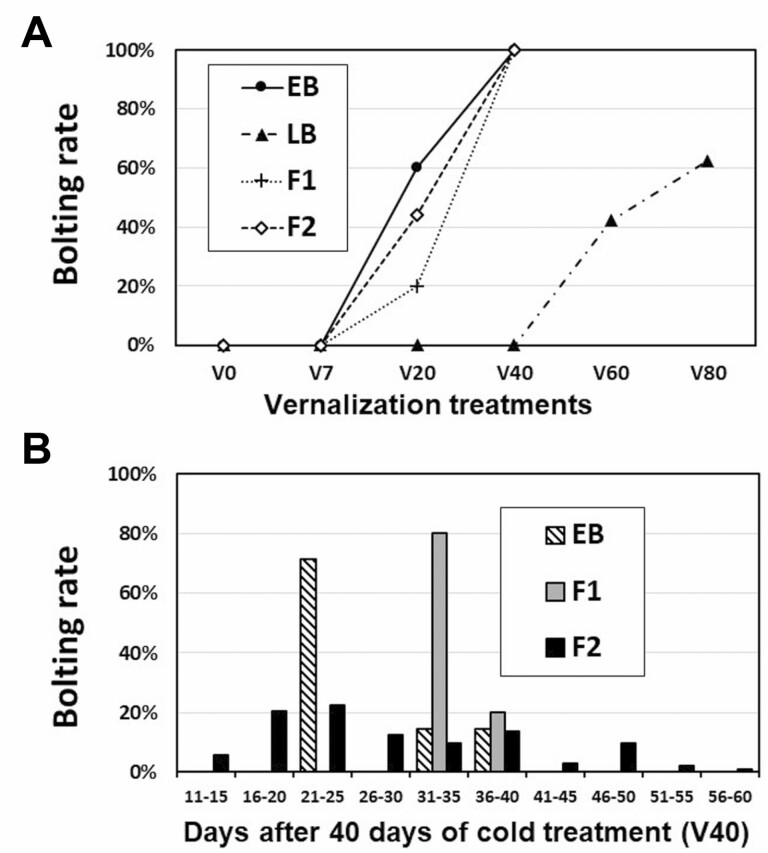
Bolting rates at (A) 60 days after the end of each vernalization treatment for EB (*n* = 30 for each treatment), extremely LB cultivar (*n* = 30), F1 population (*n* = 10) generated by interbreeding of the EB and the LB cultivars and their F2 populations (*n* = 30). (B) The number of days required for bolting after the end of 40 days of cold exposure. V0, V20, V40, V60, V80: 0, 20, 40, 60, 80 days of cold exposure, respectively.

The cold periods required for bolting differed greatly between the EB cultivar ‘Aokubinagafuto’ and extremely LB cultivar ‘Tokinashi,’ and their F1 and F2 populations showed intermediate traits (Fig. 1). Of the EB plants, 60 % had bolted by V20 (20 days of cold exposure) and all had bolted by V40 (Fig. 1A). By contrast, LB plants had bolted only by V60 and V80 (42 % and 63 %, respectively). Of the F1 and F2 plants, 20 % and 44 %, respectively, had bolted by V20 and all had bolted by V40. The periods required for bolting after the end of the V40 treatment are shown in Fig. 1B. Of the EB plants, 71 % had bolted within 25 days and all had bolted within 40 days after the end of V40 treatment. Of the F1 hybrids between EB and LB, 80 % had bolted within 35 days and all had bolted within 40 days after the end of V40 treatment. In the F2 population, the number of days to bolting after the end of V40 treatment ranged from 11 to 60, peaking at 21–25 days.

### Expression patterns of flowering time genes in EB and extremely LB radish cultivars

**Figure 2.**
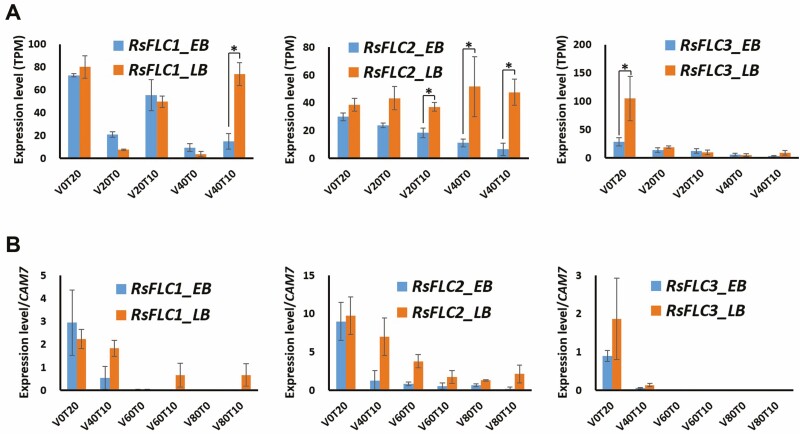
Expression levels of the three *RsFLC* genes under different vernalization conditions, measured by (A) RNA sequencing and (B) qRT-PCR. * Represents significant expression differences (Bonferroni-corrected *P*-value < 0.05) between the B and the extremely LB cultivars in each vernalization condition by RNA sequencing. V0, V20, V40, V60, V80: 0, 20, 40, 60, 80 days of cold exposure, respectively. T0, T10, T20: 0, 10, 20 days after returning to warm conditions, respectively. Error bar: standard deviation.

RNA-Seq analysis of the 280 previously reported flowering-related genes revealed that seven flowering integrator genes, five vernalization genes, nine photoperiodic/circadian clock genes and eight genes from other flowering pathways were differentially expressed in the EB and extremely LB cultivars under different vernalization conditions. In flowering integrator genes, the three copies of the *RsFLC* flowering repressors were differentially expressed (Fig. 2 and **Supporting Information—Fig. S1A**). Under 40 days of cold exposure, the three *RsFLC* copies were downregulated in EB, even after the plants were placed in a warm environment (V40T10). By contrast, only *RsFLC3* was downregulated in the LB cultivar, and no significant change in *RsFLC1* or *RsFLC2* expression from the non-vernalized level was observed under the V40T10 treatments. Interestingly, *RsFLC2* was stably expressed in the LB cultivar under all conditions, whereas *RsFLC1* expression was severely decreased under cold conditions but restored to the non-vernalization level after the LB plants were returned to warm conditions (V40T10). We also confirmed the expression levels of the three *RsFLC* genes by performing qRT-PCR analysis (Fig. 2). The expression profiles of the three *RsFLC* genes at V0T20 and V40T10 were consistent with the RNA-Seq results. In the LB cultivar, *RsFLC1* and *RsFLC2* expression levels gradually decreased after 60 and 80 days of cold exposure, respectively.

**Figure 3.**
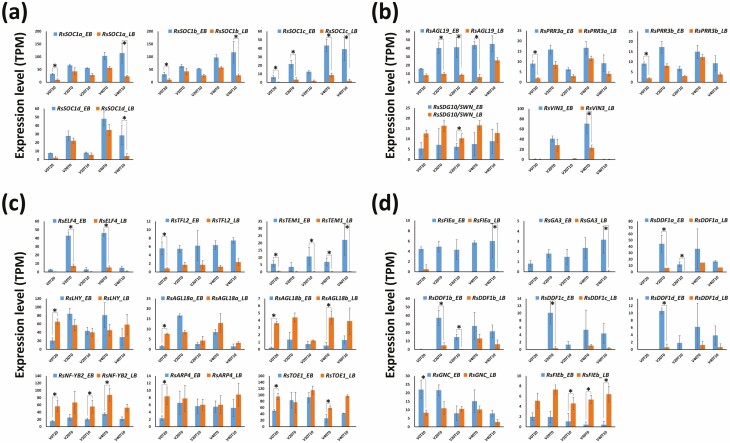
Expression levels of DEGs under different vernalization conditions, measured by RNA sequencing. (A) Flowering integrator genes (excluding *RsFLCs*), (B) vernalization pathway genes, (C) photoperiod/circadian clock pathway genes, (D) genes involved in other flowering pathways. * Represents significant differences (Bonferroni-corrected *P*-value < 0.05) between the EB and the extremely LB cultivars in each vernalization condition. V0, V20, V40: 0, 20, 40 days of cold exposure, respectively. T0, T10, T20: 0, 10, 20 days after returning to warm conditions, respectively. Error bar: standard deviation.

In flowering integrators other than the *FLC* genes, the four *RsSOC1* copies were significantly upregulated in the EB cultivar after 40 days of cold exposure, but downregulated in the LB cultivar (Fig. 3A and **Supporting Information—Fig. S1A**). The other flowering integrator genes, *RsFTa*, *RsFTb*, *RsCO* and *RsLFY*, were not differentially expressed between EB and LB although expression levels of *RsFTa* were relatively high at V40T0 and V40T10 in the EB cultivar **[see****Supporting Information—Fig. S2****]**. Five vernalization genes were differentially expressed in the EB and LB cultivars under various vernalization conditions (Fig. 3B and **Supporting Information—Fig. S1B**). Expression levels of *RsAGL19* and the two *RsPRR3* copies were relatively low, whereas *RsSDG10/SWN* expression was upregulated, in the LB cultivar. The expression of *RsVIN3* was relatively high in the EB cultivar under cold conditions, but very low under warm conditions in both the EB and LB cultivars. Nine photoperiod and/or circadian clock pathway genes were also differentially expressed in the EB and LB cultivars under each vernalization condition (Fig. 3C and **Supporting Information—Fig. S1C**). *RsELF4*, *RsTFL2* and *RsTEM1* were relatively downregulated in the LB cultivar, and the expression levels of *RsLHY* and *RsAGL18a* differed among vernalization conditions. *RsAGL18b*, *RsNF-YB2*, *RsARP4* and *RsTOE1* were relatively upregulated in the LB cultivar. Eight genes involved in other flowering pathways were differentially regulated (Fig. 3D), and their expression patterns were largely clustered in the EB and LB cultivars **[see****Supporting Information—Fig. S1D****]**. *RsFIEa*, *RsGA3*, four copies of *RsDDF1* and *RsGNC* were downregulated, whereas *RsFIEb* was upregulated, in the LB cultivar. The radish flowering pathways associated with these DEGs are summarized in **Supporting Information—Fig. S3**.

### Associations among *RsFLC1/2* genotypes, bolting times and gene expression levels

**Figure 4.**
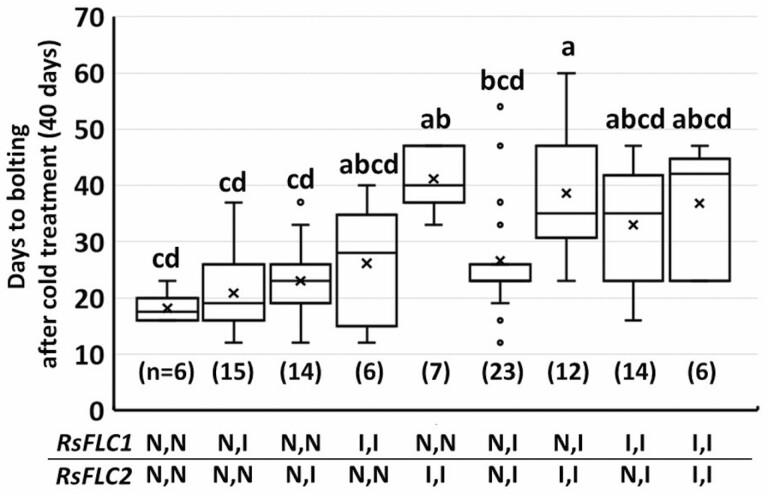
Bolting time after 40 days of cold treatment in each genotype of the F2 population generated by crossing the EB and extremely LB cultivars. I: insertion alleles with an 1910-bp or 1627-bp insertion in the first intron of *RsFLC1* or *RsFLC2*, respectively. N: non-insertion alleles without an insertion in the first intron of *RsFLC1* or *RsFLC2*. Different alphabetical characters represent significant differences (*P* < 0.05, Kruskal–Wallis/Steel–Dwass tests).

The EB cultivar was homozygous for non-insertion (N) alleles of *RsFLC1* and *RsFLC2*, which contained no insertion in the first introns, whereas the LB cultivar was homozygous for insertion (I) alleles of *RsFLC1* and *RsFLC2*. In the F2 population, 26.2 % were homozygous for N-alleles of both *RsFLC1* and *RsFLC2*, whereas 25.2 % and 24.3 % were homozygous for I-alleles of *RsFLC1* and *RsFLC2*, respectively; and 48.5 % and 49.5 % were heterozygous for *RsFLC1* and *RsFLC2* I-alleles, respectively. In the F2 population, genotypes with larger numbers of I-alleles tended to require longer periods for bolting after 40 days of cold treatment (Fig. 4). The mean and median numbers of days required for bolting were increased in homozygotes with I-alleles for *RsFLC2* or *RsFLC1*. In particular, bolting was significantly delayed for genotypes with two *RsFLC2* I-alleles and one or two *RsFLC1* N-alleles relative to genotypes in which the N-allele was dominant.

**Figure 5.**
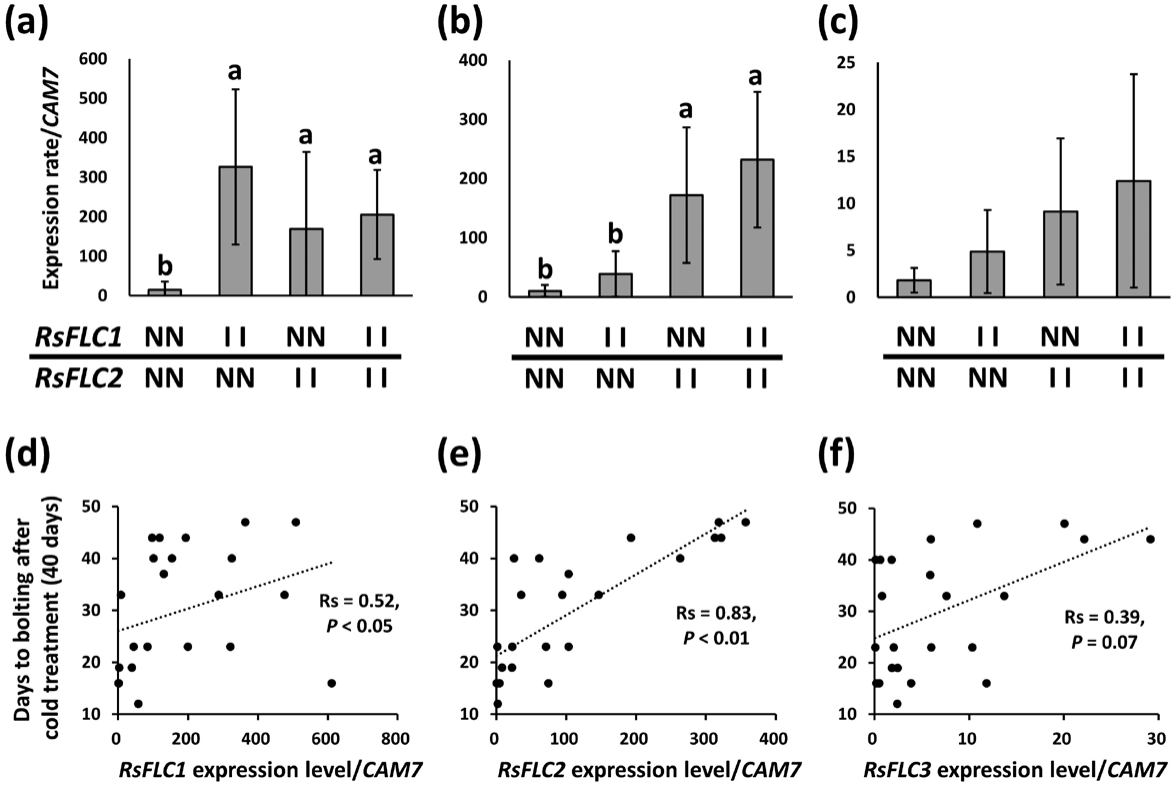
Associations of the expression levels of the three *RsFLC* genes with *RsFLC1* and *RsFLC2* genotypes and bolting times. Expression levels of (A) *RsFLC1*, (B) *RsFLC2* and (C) *RsFLC3* in the four genotypes of *RsFLC1* and *RsFLC2*. I: insertion alleles with a 1910-bp and 1627-bp insertion in the first intron of *RsFLC1* and *RsFLC2*, respectively. N: non-insertion alleles without an insertion in the first intron of *RsFLC1* or *RsFLC2*. Error bar: standard deviation. Correlations of the expression rates of (D) *RsFLC1*, (E) *RsFLC2* and (F) *RsFLC3* with the number of days to bolting after 40 days of cold treatment. *R*_s_: Spearman’s rank-order correlation coefficients.

The expression levels of the three *RsFLC* genes differed in *RsFLC1* and *RsFLC2* genotypes. After 40 days of cold exposure followed by 10 days of warm conditions, the expression levels of *RsFLC1* were significantly lower in plants homozygous for both *RsFLC1* and *RsFLC2* N-alleles than in those with I-allele genotypes (*P* < 0.01; Fig. 5A). Interestingly, homozygous *RsFLC2* I-allele genotypes with no insertion in *RsFLC1* did not exhibit decreased *RsFLC1* expression. We found a significant correlation between *RsFLC1* expression and number of days to bolting (*P* < 0.05; Fig. 5D). Two-way ANOVA also showed that *RsFLC1* expression was influenced by the *RsFLC1* genotype (*P* < 0.01; Table 1), and significant interactions between the *RsFLC1* genotypes and *RsFLC2* genotypes were detected (*P* < 0.01). The expression levels of *RsFLC2* were significantly higher in homozygotes of *RsFLC2* I-alleles (*P* < 0.01; Fig. 5B), while *RsFLC1* insertion did not maintain *RsFLC2* expression. A significant correlation between *RsFLC2* expression and number of days to bolting was also detected (*P* < 0.01; Fig. 5E). Two-way ANOVA revealed that the *RsFLC2* genotype significantly affected *RsFLC2* expression (*P* < 0.01; Table 1). *RsFLC3* expression was significantly lower than that of *RsFLC1* and *RsFLC2*. Genotypes with I-alleles tended to show higher *RsFLC3* expression levels although the difference was not significant (*P* = 0.065; Fig. 5C). We found no correlation between *RsFLC3* expression and number of days to bolting (Fig. 5F). Two-way ANOVA revealed a significant effect of the *RsFLC2* genotype on *RsFLC3* expression (Table 1). Overall, homozygosity for the I-allele, particularly in *RsFLC2*, upregulated the expression levels of all three *RsFLC* paralogs.

**Table 1.** Two-way repeated ANOVA of gene expression of *RsFLC1*, *RsFLC2* and *RsFLC3*and the genotype difference of *RsFLC1* and *RsFLC2*. N: non-insertion allele, I: insertion allele.

Source	df	Mean square	*F*	*P*
*RsFLC1* expression				
* RsFLC1* genotype (NN or II)	1	4.782 × 10^5^	18.470	<0.01
* RsFLC2* genotype (NN or II)	1	4.804 × 10^3^	0.158	0.690
* RsFLC1* genotype × *Rs FLC2* genotype	1	2.941 × 10^5^	11.358	<0.01
* *Error	44	2.589 × 10^4^		
*RsFLC2* expression				
* RsFLC1* genotype (NN or II)	1	3.261 × 10^4^	1.823	0.184
* RsFLC2* genotype (NN or II)	1	6.131 × 10^5^	34.271	<0.01
* RsFLC1* genotype × *Rs FLC2* genotype	1	1.519 × 10^3^	0.085	0.772
* *Error	44	1.789 × 10^4^		
*RsFLC3* expression				
* RsFLC1* genotype (NN or II)	1	1.726	1.919	0.173
* RsFLC2* genotype (NN or II)	1	9.829	10.928	<0.01
* RsFLC1* genotype × *Rs FLC2* genotype	1	2.592	0.003	0.955
* *Error	44	8.994		

To investigate the origins and/or sequence homologies of the intron insertions of *RsFLC1* and *RsFLC2*, we conducted BLASTn and BLASTX searches of the inserted sequences against the National Center for Biotechnology Information non-redundant database using e-100 as the significance similarity threshold **[see****Supporting Information—Table S3****]**. Highly similar sequences of the 1910-bp insertion in the first intron of *RsFLC1* were found in the chromosome 4, 8 and 9 in the radish genome assembly using BLASTn. The 1910-bp insertion in the first intron of *RsFLC1* shared homologies with the *B. nupus*, *B. oleracea* and *B. rapa* genome assemblies, with query coverages of 51.5–57.0 %. However, no similar genes were found using BLASTX. In the BLASTn search, the 1627-bp insertion in the first intron of *RsFLC2* shared homologies with the radish chromosome 1, 3 and 9, with query coverages of 68.1–99.2 %, and the *B. nupus* and *B. oleracea* genome assemblies, with query coverages of 69.0–71.9 %; no similar genes were found by BLASTX. No homologous sequence of transposable elements was detected for both *RsFLC1* and *RsFLC2* insertions.

## Discussion

### Differential expression of flowering-related genes between the EB and LB cultivars

Our transcriptome analysis identified several DEGs involved in various flowering pathways between the EB cultivar ‘Aokubinagafuto’ and LB cultivar ‘Tokinashi’ under vernalization treatments. The expression patterns of flowering integrator genes, including *RsFLC1* and *RsFLC2*, which are flowering repressors, and the four *RsSOC1* copies, which are floral inducers, differed greatly between the EB and LB cultivars, suggesting that these expression differences are involved in flowering time variation. Signals from different flowering pathways are integrated by the floral inducers FT, SOC1 and LFY, leading to bolting and flowering (Moon *et al.* 2005; Lee and Lee 2010; Srikanth and Schmid 2011). SOC1 belongs to the MADS-box family and directly promotes flowering and *LFY* expression (Lee *et al.* 2008; Seo *et al.* 2009; Lee and Lee 2010). In the EB cultivar, expression levels of the four *RsSOC1* paralog copies were upregulated after 40 days of cold exposure and subsequent warm periods. In the LB cultivar, the expression levels of *RsFLC1* and *RsFLC2* did not decrease in subsequent warm conditions after 40 days of cold exposure, and *RsSOC1* expression was repressed. These results indicate that the stable expression of *RsFLC1* and *RsFLC2* in the LB cultivar under cold conditions and subsequent warm periods may be a cause of late flowering by suppressing the expression of *RsSOC1*. FLC is the main flowering repressor that integrates signals from the vernalization and autonomous pathways, and it negatively regulates the expression of floral inducers (Helliwell *et al.* 2006; Seo *et al.* 2009). Several functional *FLC* paralogs are present in Brassicaceae family members, including radish (Yi *et al.* 2014) and *Brassica* (Kim *et al.* 2007). Thus, the flowering time of Brassicaceae crops is considered to be regulated by multiple *FLC* genes.

We found that five vernalization response genes were differentially expressed between the EB and LB cultivars. Among these, *AGAMOUS-LIKE 19* (*RsAGL19*) expression levels were highly upregulated in the EB cultivar compared to the LB cultivar in several vernalization conditions. *AGL19* is a MADS-box gene repressed by polycomb-group proteins under non-cold conditions; however, its expression is accelerated by prolonged cold, resulting in the promotion of flowering (Schönrock *et al.* 2006). Prolonged cold relieves *AGL19* from polycomb-group protein repression via a mechanism involving VIN3 and elevated AGL19 activates *LFY* and *AP1* as part of the poorly characterized FLC- and SOC1-independent vernalization pathway (Schönrock *et al.* 2006). We found that *RsVIN3* expression level was relatively lower in the LB cultivar than in the EB cultivar under cold conditions.. These insights suggest that AGL19 could be mediating a signalling pathway acting independently and that differential expression of this vernalization pathway may contribute to flowering time variation among radish cultivars. VIN3 also participates in the suppression of *FLC* under cold conditions, but is not required for *FLC* repression maintenance under warm conditions after a cold period (Gendall *et al.* 2001; Sung and Amasino 2004). Consistent with previous studies, we found that *RsVIN3* was upregulated only under cold conditions.

Day length (photoperiod) is another major environmental factor that affects the timing of floral transition. CONSTANS (CO) plays a central role in the photoperiod pathway, and its expression is controlled by the circadian clock and light signals (Putterill *et al.* 1995; Suárez-López *et al.* 2001; Searle and Coupland 2004). Under long-day conditions, *FT* expression is activated by CO (Amasino and Michaels 2010). Our transcriptome analysis revealed nine DEGs involved in the photoperiod and circadian clock pathways; however, *CO* was not differentially expressed and its expression was detected at low levels in both cultivars. The expression of *RsFT* genes also did not differ significantly between the EB and LB cultivars, although previous studies found that these genes were upregulated during floral transition in radish (Nie *et al.* 2016b). The expression dynamics of *RsFT* and *RsFLC* differ throughout the circadian cycle (Han *et al.* 2021); *RsFLC* expression peaks at the beginning of the day, or at Zeitgeber time (ZT) 4–8, where ZT represents a standard 24 h notation representing the phases of an entrained circadian cycle. By contrast, *RsFT* expression peaks later in the day, at approximately ZT 16 (Han *et al.* 2021). In this study, we collected leaves and extracted RNA at approximately ZT 6, such that low *RsFT* expression was detected. We found several DEGs in the circadian clock and photoperiodic pathways; however, their roles in flowering time variation remain unknown due to the low expression levels for the integrator genes *RsCO* and *RsFT*.

Among gibberellin pathway genes, *RsGA3*, four copies of *RsDDF1* and *RsGNC* were downregulated in the LB cultivar. These genes are thought to be involved in gibberellin biosynthesis (Helliwell *et al.* 1999; Magome *et al.* 2004; Richter *et al.* 2013). LFY, the floral integrator, functions mainly in the shoot apex, and LFY expression is tightly regulated by endogenous gibberellic acid levels (Yu *et al.* 2002; Eriksson *et al.* 2006). Neither *RsLFY* gene was expressed in leaves collected in this study. *RsLFY* expression has been detected in radish flower organs (Nie *et al.* 2016b), suggesting that radish LFY also functions in flower organs. Delayed flowering in the LB cultivar may be due partly to the downregulation of gibberellin biosynthesis. Further study of the accumulation of endogenous gibberellins and *RsLFY* expression patterns in floral organs is needed.

### *RsFLC1/2* genotype bolting times and gene expression levels

Association analyses using an EB × LB F2 population, in which the insertions in the first introns of *RsFLC1* and *RsFLC2* were segregated, revealed that these insertions caused expression changes in the *RsFLC* genes, leading to late flowering. A previous inheritance analyses using F2 and a population obtained by backcrossing the EB and LB radish cultivars suggested that two major genes with additive and epistatic effects are involved in flowering time variation (Wang *et al.* 2018a). Furthermore, they found that an expressed sequence tag-simple sequence repeat (EST–SSR) marker associated with flowering time was in the same linkage group as *RsFLC2*, although they could not identify the gene due to the limited number of markers (Wang *et al.* 2018a). A QTL analysis using 500 indel markers in the EB cultivar and the LB cultivar ‘Ninengo’ revealed that *RsFLC2* is associated with the LB trait, and that the allele with a 1627-bp insertion in the first intron, the same as in the ‘Tokinashi’ cultivar examined in our study, weakened the repression of *RsFLC2* expression (Wang *et al.* 2018b). Our results are consistent with previous findings, suggesting that *RsFLC2* and *RsFLC1* are the major genes responsible for late-flowering traits. In particular, we found that the insertion in the first intron of *RsFLC2* is correlated with the downregulation of the other *RsFLC* genes during vernalization and late flowering. Since the *RsFLC1* insertion allele affected expression of itself only, vernalization sensitivities may differ between the insertion sites and sequences. In *B. rapa*, it was hypothesized that *BrFLC2*, the ortholog of *RsFLC2* (Yi *et al*. 2014), is a major candidate vernalization response regulator based on QTL analyses (Lou *et al.* 2007; Zhao *et al.* 2010; Kitamoto *et al.* 2014). Collectively, these insights and our results indicate that FLC2 proteins in *B. rapa* and radish play central roles in flowering time regulation. The *RsFLC1* and *RsFLC2* insertions exhibited homology with non-gene sequences of some *Brassica* species, suggesting that these sequences are shared between LB *Brassica* and radish strains. On the other hand, a previous study found that a ~5 kb insertion in the first introns of *BrFLC2* and *BrFLC3*, which contributed to late-flowering traits, shared sequence homology with non-long terminal repeat retroelement reverse transcriptase and putative reverse transcriptase genes from *A. thaliana* (Kitamoto *et al.* 2014). Thus, several types of insertions of various origins are likely responsible for late-flowering traits.

The first intron of *FLC*, in addition to its promoter and exon 1, contains important elements that are crucial for the cold-induced repression of *FLC* and maintenance of its repression after returning to warm conditions (Sheldon *et al.* 2002). Variation in vernalization sensitivity is caused by various indel mutations in the first intron of *FLC* in *Arabidopsis* (Michaels *et al.* 2003; Wang *et al.* 2006; Strange *et al.* 2011), suggesting that multiple *cis*-elements are present in the first *FLC* intron. A 289-bp deletion of the VRE in the first intron of *FLC* weakened *FLC* repression during cold exposure and after returning to warm conditions (Sung *et al.* 2006). In addition, the intronic lncRNA, *COLDAIR*, is transcribed from the VRE and is involved in polycomb-mediated epigenetic silencing of *FLC* (Heo and Sung 2011). *COLDAIR* is temporally expressed, peaking at approximately 20 days of coldness. It physically associates with a component of PRC2, which it targets to the first intron of *FLC* (Heo and Sung 2011; Ietswaart and Dean 2012; Kim and Sung 2017). This leads to histone deacetylation and methylation of H3K9 and H3K27, which maintain *FLC* repression at warm temperatures after cold exposure (Sheldon *et al.* 2002; Sung and Amasino 2004). The VRE is located in the 5ʹ region of the first intron and is evolutionally conserved in *Arabidopsis* and related species (Castings *et al.* 2014). In this study, we found sequences homologous to the *Arabidopsis* VRE in the first intron of radish *FLC* genes **[see****Supporting Information—Fig. S4****]**. Insertions were present in *RsFLC1* and *RsFLC2* close to the 5ʹ region of VRE-like conserved sequences, suggesting that these insertions decreased the vernalization sensitivity of these genotypes, perhaps by inhibiting the binding of chromatin modification factors and/or by suppressing *COLDAIR* transcription. During the early stages of vernalization, before the expression of *COLDAIR*, a set of alternatively spliced, polyadenylated antisense RNAs, named *COOLAIR*, is transiently expressed from the 3ʹ end of *FLC* to its promoter, spanning exon 1 and the 5ʹ end of the first intron (Liu *et al.* 2010). *COOLAIR* may be involved in vernalization-mediated *FLC* repression (Swiezewski *et al.* 2009; Liu *et al.* 2010; Heo and Sung 2011). Furthermore, *COOLAIR* participates in the acceleration of *FLC* repression during vernalization, independently of PRC2 and H3K27me3 (Csorba *et al.* 2014). *ANTISENSE LONG* (*ASL*) is another antisense transcript originating from the 3ʹ end of *FLC* and spanning to the first intron (Shin and Chekanova 2014). The *ASL* transcript physically associates with the *FLC* locus and H3K27me3 (Shin and Chekanova 2014). Thus, the first intron of *FLC* contains *cis*- and *trans*-elements that play pivotal roles in *FLC* regulation. A recent study showed that vernalization treatment induces *COOLAIR* homologues in *B. rapa* (Shea *et al.* 2019). Expression of the *COOLAIR* homologues has also been detected by transcriptome analysis of lncRNA and 5ʹ and 3ʹ rapid amplification of cDNA ends (RACE) analyses in radish (Y. Mitsui *et al.*, unpubl. data). These insights suggest that functional interactions occur among radish *FLC* paralogs, possibly via *trans*-acting lncRNAs. Additional investigations into histone modifications and the identification and expression of lncRNAs are needed to elucidate the functions of the *FLC* insertion sites.

## Conclusion

In this study, we aimed to delineate the possible mechanisms responsible for late-flowering traits in radish by identifying candidate genes and analysing the relationships among allelic variation, gene expression patterns and bolting time. Our transcriptome analysis revealed that seven flowering integrator genes, five vernalization genes, nine photoperiodic/circadian clock genes and eight genes from other flowering pathways were differentially expressed in the EB cultivar ‘Aokubinagafuto’ and LB cultivar ‘Tokinashi’, under different vernalization conditions. Among the flowering integrator genes, the four *RsSOC1* copies, which are flowering inducers, were downregulated and the three copies *RsFLC* copies, which are flowering repressors, were upregulated in the LB cultivar compared to the EB cultivar in the vernalization conditions. Subsequent analyses of EB × LB F2 populations showed that each of the two insertions in the first introns of *RsFLC1* and *RsFLC2* was considered to be contributed to the late-flowering trait via different mechanisms. In particular, the *RsFLC2* insertion allele strongly delayed bolting by inhibiting the repression of all three *RsFLC* gene paralogs. Our results suggest that the flowering time of radish is determined by epistatic interactions among replicated *FLC* genes. This provides a unique system for investigating regulatory interactions among evolutionary divergent loci originating from orthologous genomes. As the same *RsFLC1* and *RsFLC2* insertion alleles are found in several radish cultivars, our results will be helpful for further breeding improvements.

## Supplementary Material

plac066_suppl_Supplementary_Figure_S1

plac066_suppl_Supplementary_Figure_S2

plac066_suppl_Supplementary_Figure_S3

plac066_suppl_Supplementary_Figure_S4

plac066_suppl_Supplementary_Table_S1

plac066_suppl_Supplementary_Table_S2

plac066_suppl_Supplementary_Table_S3

## Data Availability

The data set supporting the conclusions of this article is available in the DDBJ Sequence Read Archive (https://ddbj.nig.ac.jp/DRASearch/, accession number DRA011999).

## Notes

**Phenome, Genome & Environment. Chief Editor:** Colleen Doherty
