# Supplementary figures and images for: Epistatic interactions among multiple copies of FLC genes with naturally occurring insertions correlate with flowering time variation in radish
Source: AoB Plants. 2023 Feb 2;15(2):plac066. doi: 10.1093/aobpla/plac066 (PMC9893874; doi:10.1093/aobpla/plac066)

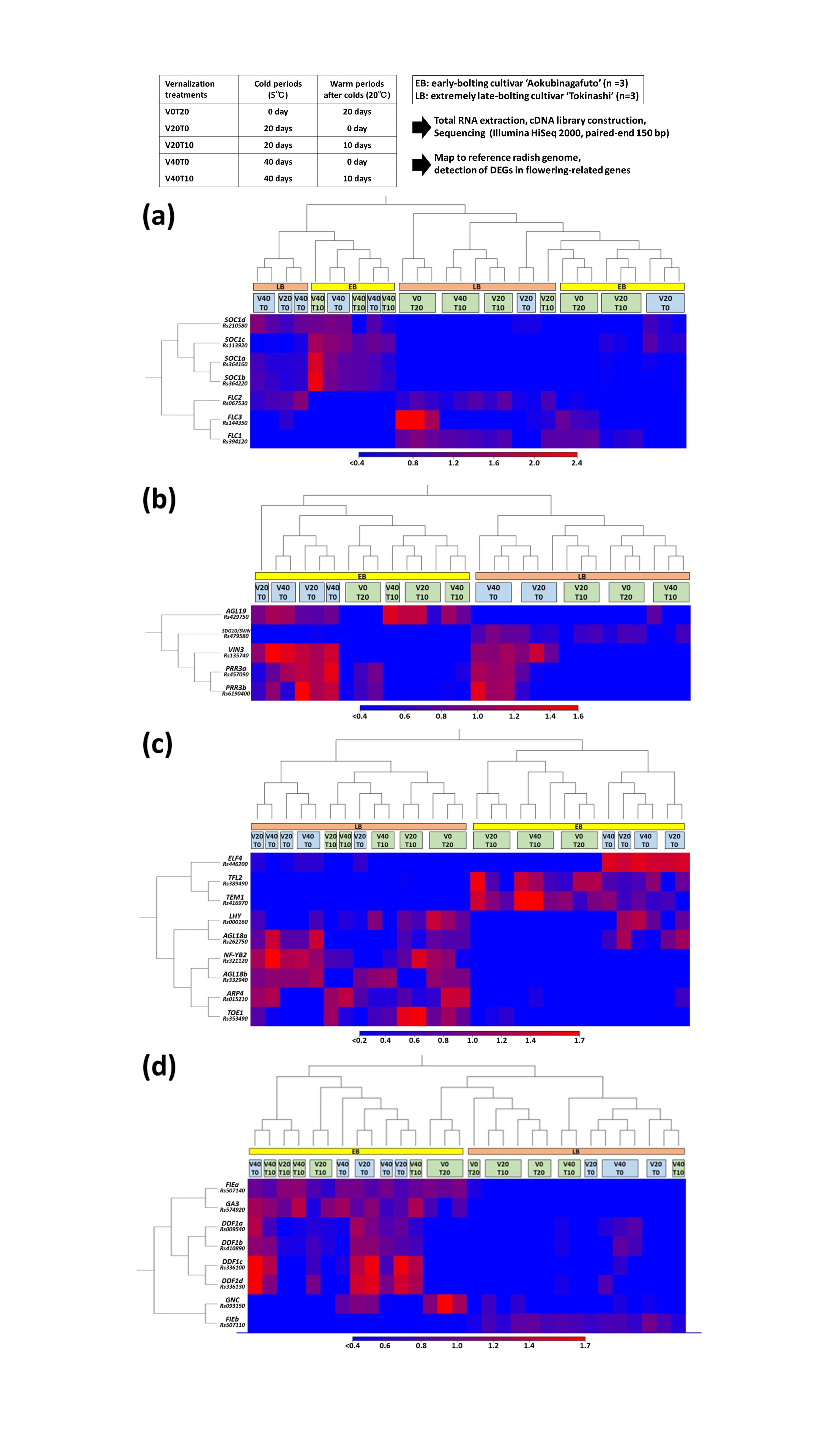

Supplement: plac066_suppl_Supplementary_Figure_S1 [file plac066_suppl_supplementary_figure_s1.jpeg]

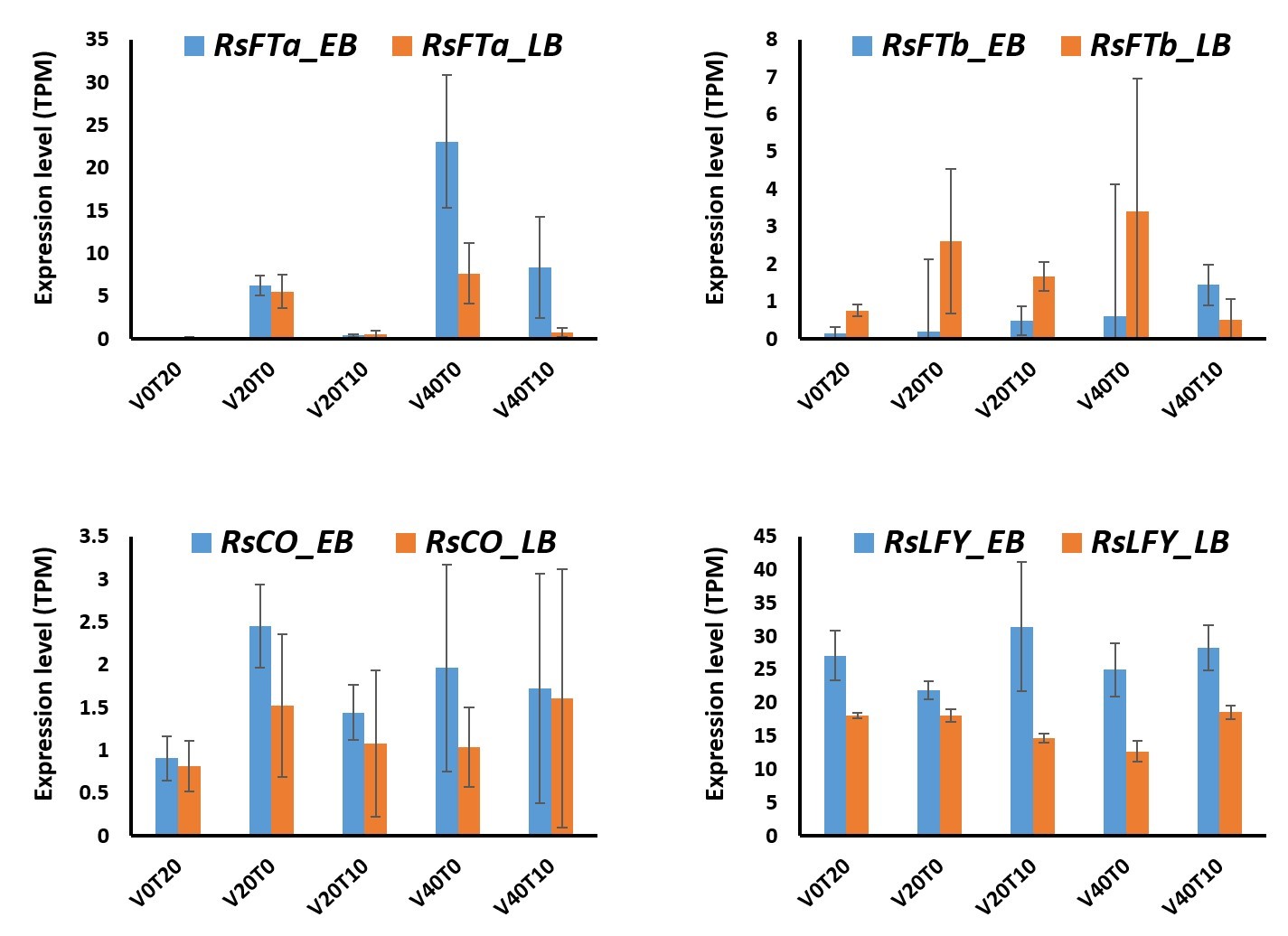

Supplement: plac066_suppl_Supplementary_Figure_S2 [file plac066_suppl_supplementary_figure_s2.jpeg]

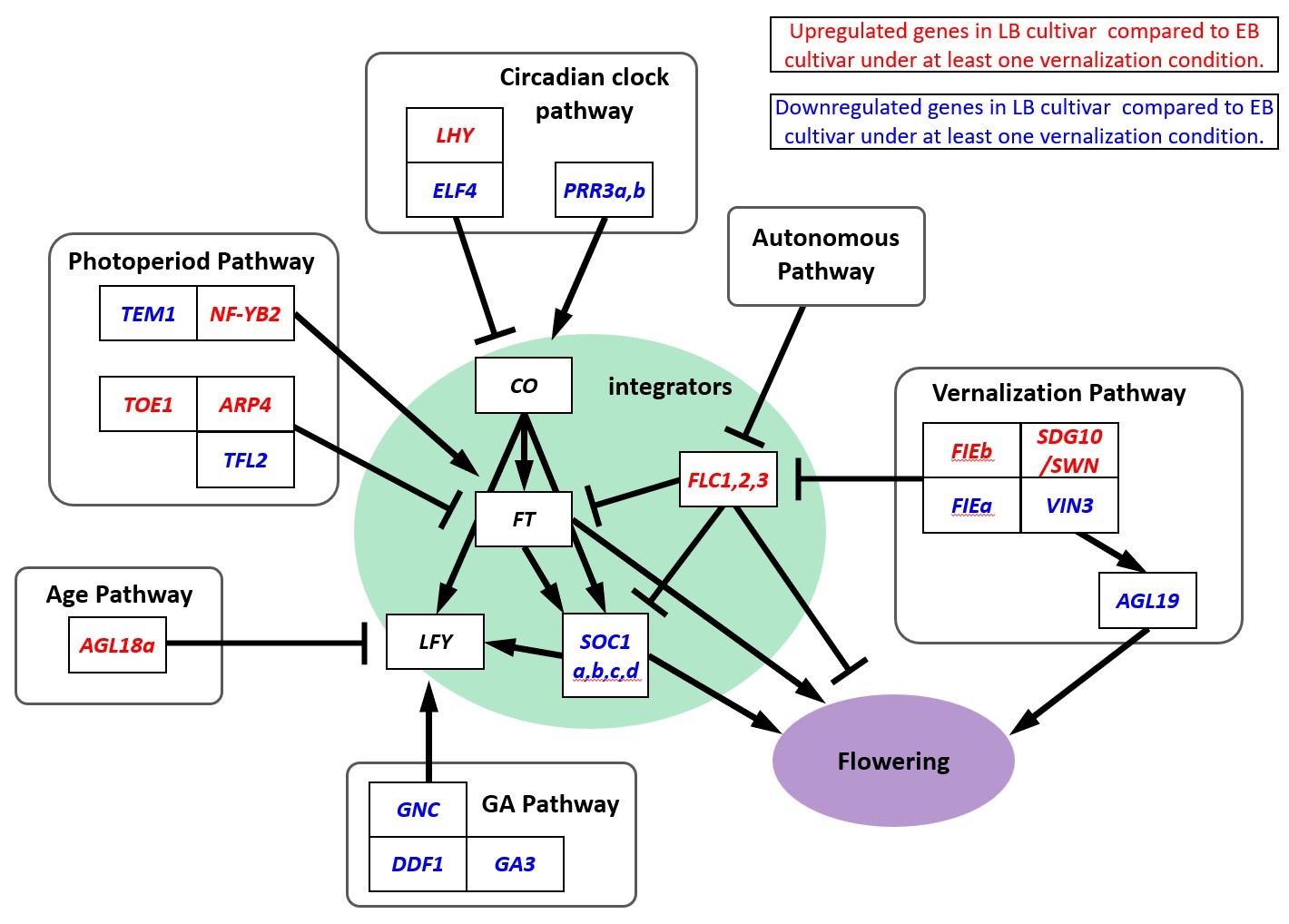

Supplement: plac066_suppl_Supplementary_Figure_S3 [file plac066_suppl_supplementary_figure_s3.jpeg]

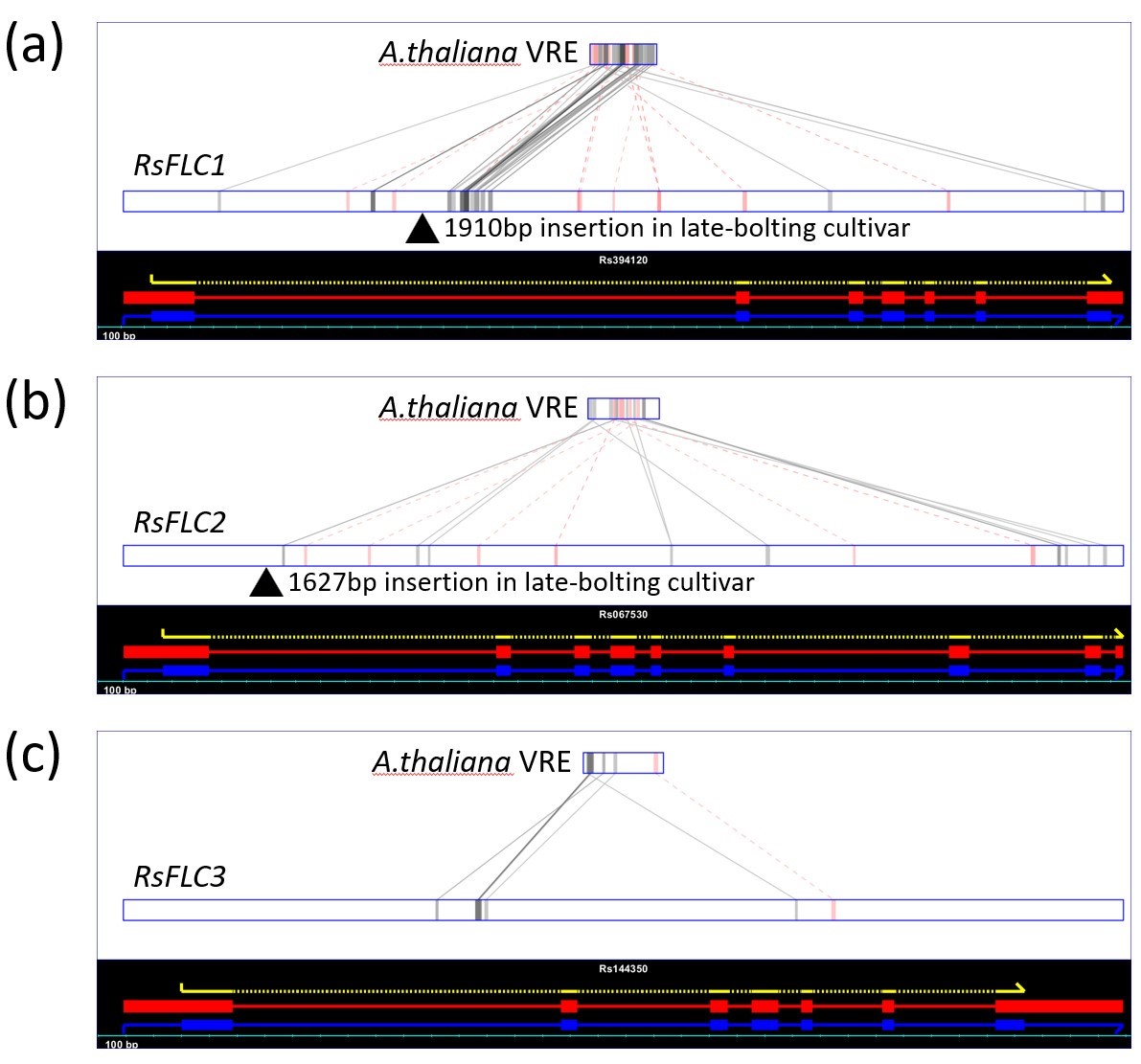

Supplement: plac066_suppl_Supplementary_Figure_S4 [file plac066_suppl_supplementary_figure_s4.jpeg]
